# Nomenclature of the symptoms of head and neck cancer: a systematic scoping review

**DOI:** 10.3389/fonc.2024.1404860

**Published:** 2024-06-17

**Authors:** Paula T. Bradley, Ying Ki Lee, Abigail Albutt, John Hardman, Ian Kellar, Chinasa Odo, Rebecca Randell, Nikki Rousseau, Theofano Tikka, Joanne M. Patterson, Vinidh Paleri

**Affiliations:** ^1^ Population Health, Newcastle University, Newcastle upon Tyne, United Kingdom; ^2^ Department of Otolaryngology, Guy’s and St Thomas’ National Health Service (NHS) Foundation Trust, London, United Kingdom; ^3^ Faculty of Medicine and Health, University of Leeds, Leeds, United Kingdom; ^4^ Department of Otolaryngology, Barts Health NHS Trust, London, United Kingdom; ^5^ Department of Psychology, The University of Sheffield, Sheffield, United Kingdom; ^6^ Faculty of Health Studies, University of Bradford, Bradford, United Kingdom; ^7^ Department of Otolaryngology, Cambridge University Hospitals NHS Foundation Trust, Cambridge, United Kingdom; ^8^ School of Allied Health Professions & Nursing, Institute of Population Health / Liverpool Head and Neck Centre, University of Liverpool, Liverpool, United Kingdom; ^9^ Head and Neck Unit, Royal Marsden NHS Foundation Trust, London, United Kingdom

**Keywords:** head and neck cancer, symptom assessment, risk assessment scoring system, gatekeeper, referral

## Abstract

**Introduction:**

Evolution of a patient-reported symptom-based risk stratification system to redesign the suspected head and neck cancer (HNC) referral pathway (EVEREST-HN) will use a broad and open approach to the nomenclature and symptomatology. It aims to capture and utilise the patient reported symptoms in a modern way to identify patients’ clinical problems more effectively and risk stratify the patient.

**Method:**

The review followed the PRISMA checklist for scoping reviews. A search strategy was carried out using Medline, Embase and Web of Science between January 1st 2012 and October 31st 2023. All titles, abstracts and full paper were screened for eligibility, papers were assessed for inclusion using predetermined criteria. Data was extracted pertaining to the aims, type of study, cancer type, numbers of patients included and symptoms, presenting complaints or signs and symptoms.

**Results:**

There were 9,331 publications identified in the searches, following title screening 350 abstracts were reviewed for inclusion and 120 were considered for eligibility for the review. 48 publications met the eligibility criteria and were included in the final review. Data from almost 11,000 HNC patients was included. Twenty-one of the publications were from the UK, most were retrospective examination of patient records. Data was extracted and charted according to the anatomical area of the head and neck where the symptoms are subjectively and objectively found, and presented according to lay terms for symptoms, clinical terms for symptoms and the language of objective clinical findings.

**Discussion:**

Symptoms of HNC are common presenting complaints, interpreting these along with clinical history, examination and risk factors will inform a clinician’s decision to refer as suspected cancer. UK Head and Neck specialists believe a different way of triaging the referrals is needed to assess the clinical risk of an undiagnosed HNC. EVEREST-HN aims to achieve this using the patient history of their symptoms. This review has highlighted issues in terms of what is considered a symptom, a presenting complaint and a clinical finding or sign.

## Introduction

Head and neck cancer (HNC) are a group of cancers affecting the head and neck including the nose, mouth, throat, voice box (larynx), and salivary glands. HNC presents in multiple anatomical sites and tissue types because of this it presents with; physical changes which are noticed by a patient and objectively detectable like an ulcer or a neck lump, symptoms like pain or discomfort which a patient experiences (locally or referred), altered function of the voice, swallowing, or breathing or a combination of physical signs, symptoms and change in function. Some of the symptoms of HNC are very commonly experienced in the general population, like an earache, a sore throat or hoarseness. In health systems where clinical gatekeepers act to determine access to clinical specialist services, decisions about who and how urgently to refer patients relies on the assessment by primary care clinicians (GP, general dental practitioners (GDP) and allied clinical staff with referral privileges). Currently there is no screening test to detect, or risk stratify suspected cases of HNC, instead patients are referred for diagnosis based on presenting signs and symptoms often with no objective clinical findings because sites are out view of examining clinicians (pharynx, larynx) or in areas with which they have little clinical experience (oral cavity). EVEREST-HN (1) aims to develop and assess the success of a system to harness responses from the patient who has been referred as a suspected HNC, to standardised questions to establish the risk of undiagnosed HNC. It is hoped that this will be suitable to help triage referrals so that specialists can better direct the patient to initial investigations, most suitable clinical assessment and determine the urgency of that assessment.

Amongst countries with a gatekeeper to specialist referrals (half of Europe, Australia, New Zealand and Canada) only the UK, Denmark, Sweden, Norway, Spain, New Zealand and Australia have guidelines for organ specific suspected cancer referrals (2, 3) including suspected HNC (4). One Danish centre (Odense) reports a conversion rate from referral to cancer diagnosis as 29.2% (1 July 2012 to 1st September 2015) (5). In the UK referral to cancer diagnosis conversion rates for head and neck cancer, reported by multiple audits of specialist centres, commonly fall below 10% (6). The suspected cancer referral to diagnosis conversion rates for head and neck cancers in the UK is one of the lowest amongst the country’s organ specific suspected cancer referral pathways despite the changes to the clinical referral criteria since 2000 (7). The most recent version of the English suspected HNC referral guidelines, determined by the National Institute of Clinical Excellence (NICE NG12) (now nearly a decade old), has been implemented in some but not all the cancer alliances in England.

In the UK the where referrals for suspected cancer come from primary care, the changing nature of the workforce, issues with patient access to dentistry and the cancer agenda set by the NHS are all drivers to the increase in suspected HNC referral volumes, which now stands at just over 200,000 referrals per annum (8). Problems peculiar to the NHS derive from government determined clinical targets and a commitment to prioritise early cancer detection (because of comparatively poor outcomes when compared to those of similarly socioeconomic status countries). In the UK this means suspected cancer referrals come without financial tariff for primary care (unlike referrals for specialist opinion about clinical issues not associated with a cancer suspicion), but targets and fines are imposed on secondary care if they miss time to diagnosis targets. This places a huge burden when it comes to delivery of outpatient services and diagnostic imaging on specialist services.

Referrals to suspected HNC services depends on a primary care clinician’s interpretation of the patient history, along with risk factors and clinical assessment. The difficulty for primary care in recognising HNC exist in large part because they will see a case of HNC so rarely, HNC originates from multiple tissue types across neighbouring anatomical sites which are often outside the examination field without specialist equipment and cancers can cause overlapping symptoms. The interpretation and misinterpretation of, particularly, the symptoms and physical signs of HNC have been a source of frustration for Ear Nose and Throat (ENT) and maxillofacial surgeons (OMFS) alike (9). The comparative ease with which the Two week wait (TWW) and Urgent Suspicion of Cancer (USOC) pathway (in Scotland) can be accessed in primary care for referrals and the emphasis on early cancer recognition and detection issued from the NHS, government and charitable bodies has decidedly changed the referral practices and subsequently resources required to meet the volume of patient referrals.

A recent publication of the analysis of UK General Practice (GP) Cancer Diagnosis Audit in 2018, exploring the signature symptoms of incident cancers, found the signature symptoms for HNC were neck lump, ulceration and, hoarseness (10). The audit was retrospective, making it subject to recall bias, and those submitting data had access to the full medical records; hence there is potential for diagnostic findings to be declared as the first symptom prompting suspicion of cancer and possibly not reflective of the true clinical course from first presentation in primary care to diagnosis in secondary care.

Much retrospective work has been conducted since the introduction of the suspected cancer pathway by UK head and neck specialists (6). Published audits examine compliance with referral guidelines and consider ways which might reduce the volume of referrals whilst maintaining the yield of cancer from the referrals. The most notable contribution to this debate is the HaNC-RC (head and neck cancer – risk calculator) which was proposed initially as a tool to be used in primary care to help determine the route of referral for patients with symptoms considered suspicious for HNC (11–13).

Covid-19 and its consequences on healthcare delivery offered an opportunity for ENT surgeons to use HaNC-RC tool to triage the suspected HNC referrals (14). This ENT UK endorsed project demonstrated that secondary care could successfully triage over the telephone using the risk calculator as part of a consultation. Those specialists involved found this an effective way to determine the risk of an undiagnosed HNC, reduce patient anxiety and arrange appropriate investigations ahead of a time appropriate face to face assessment (15). As services opened up as lockdown restrictions relaxed telephone triage was gradually phased out and so a new solution to a long term problem was sought.

EVEREST-HN takes a multidisciplinary approach and includes public and patient involvement (from inception to completion) and will use a broad and open approach to the nomenclature and symptomatology aiming to capture and utilise the patient’s responses to questions posed in appropriate language. The aim is to identify patients’ clinical problems more effectively and risk stratify, rather than by way of a simple box tick on a referral form. The hope is that it will necessitate a move away from an automatic one size fits all specialist out-patient appointment within two weeks.

## Aims of scoping review

The aim of the scoping review was to produce a comprehensive collection of the terminology related to the symptoms associated with head and neck patient presentation to help inform the development of the EVEREST-HN patient facing platform.

This scoping review will explore the literature to 1) identify the symptoms associated with the presentation of HNC, 2) provide a collection of terms and identify the source of this language to describe the patient experience of symptoms which could be caused by an undiagnosed HNC.

Scoping reviews allow an exploration of the available literature in a complex area which has perhaps not been comprehensively reviewed, they enable mapping of the main sources and available evidence about a topic (16).

The review is not concerned with risk factors of HNC nor does it not aim to assess sensitivity of the symptoms as this was done in the development of the HaNC-RC and its use during Covid-19, which has informed the development of the EVEREST-HN project and is already in the public domain.

## Method

The review is reported in accordance with the Preferred Reporting Items for Systematic Reviews and Meta-Analysis (PRISMA) extension for Scoping Reviews (PRISMA-ScR) (17). A protocol was developed by PB with agreement from VP and JP. The anticipated heterogenous nature of the data (a large proportion of the literature was not research but reports of audit and reports of retrospective patient note review) meant that there was no intention to critically appraise the publications.

The lead researcher (PB) and an Information Specialist developed a search strategy for electronic databases using MeSH terms (see **Appendix** for search strategies). An electronic search for relevant published material was done using Medline, Embase and Web of Science between January 1^st^ 2012 and October 31^st^ 2023. The only limits on the searches were that they were dated after 1^st^ January 2012 (pilot searches over a decade found more results than could be managed in the available time and resources). Publications included those which were considered quantitative, qualitative, or mixed methods, as not all publications could be regarded as empirical studies, it was decided that audits, surveys, and descriptive cohort publications could be included as this was a scoping not a systematic review or meta-analysis. Reviews, commentaries, conference abstracts and editorials were excluded but foreign language papers were not excluded from the searches.

Search results were saved into an Excel^®^ (Microsoft^®^ 365) spreadsheet and references downloaded to EndNote ™ citation manager (Clarivate) and duplicates were removed.

All titles identified in the electronic database searches were screened by PB with 10% of the titles checked by YL, PB reviewed all selected abstracts with a 10% check by YL again. Any decisions about inclusion which were not in agreement were discussed and reviewed. The full papers were obtained for all abstracts considered eligible and both PB and YL reviewed all full papers for inclusion using the predetermined criteria, any disagreements at any point in the process were discussed between PB and YL until agreement was reached.

Papers were included which had information about a cohort or case series of HNC patients with terms/vocabulary/language pertaining to their symptoms, signs, or presenting complaints, no limits were applied in terms of type of health care system (public, private or gatekeeper system with specific referral criteria), following full paper selection, PB extracted data using criteria agreed by PB and YL.

Data was extracted from the articles about the aims, type of study, cancer type, numbers of patients included along with patients’ symptoms and presenting complaints or signs and symptoms pertinent to referral criteria, YL checked the data extraction table completed by PB against the full papers and any additions or corrections were agreed between PB and YL.

Once the data was extracted and charted, the main author summarised the results in a narrative synthesis and categorised the symptoms, signs, lay and medicalised language of the presenting symptoms, complaints, and signs of HNC in a table according to anatomical functional sites.

## Results

**Figure 1:**
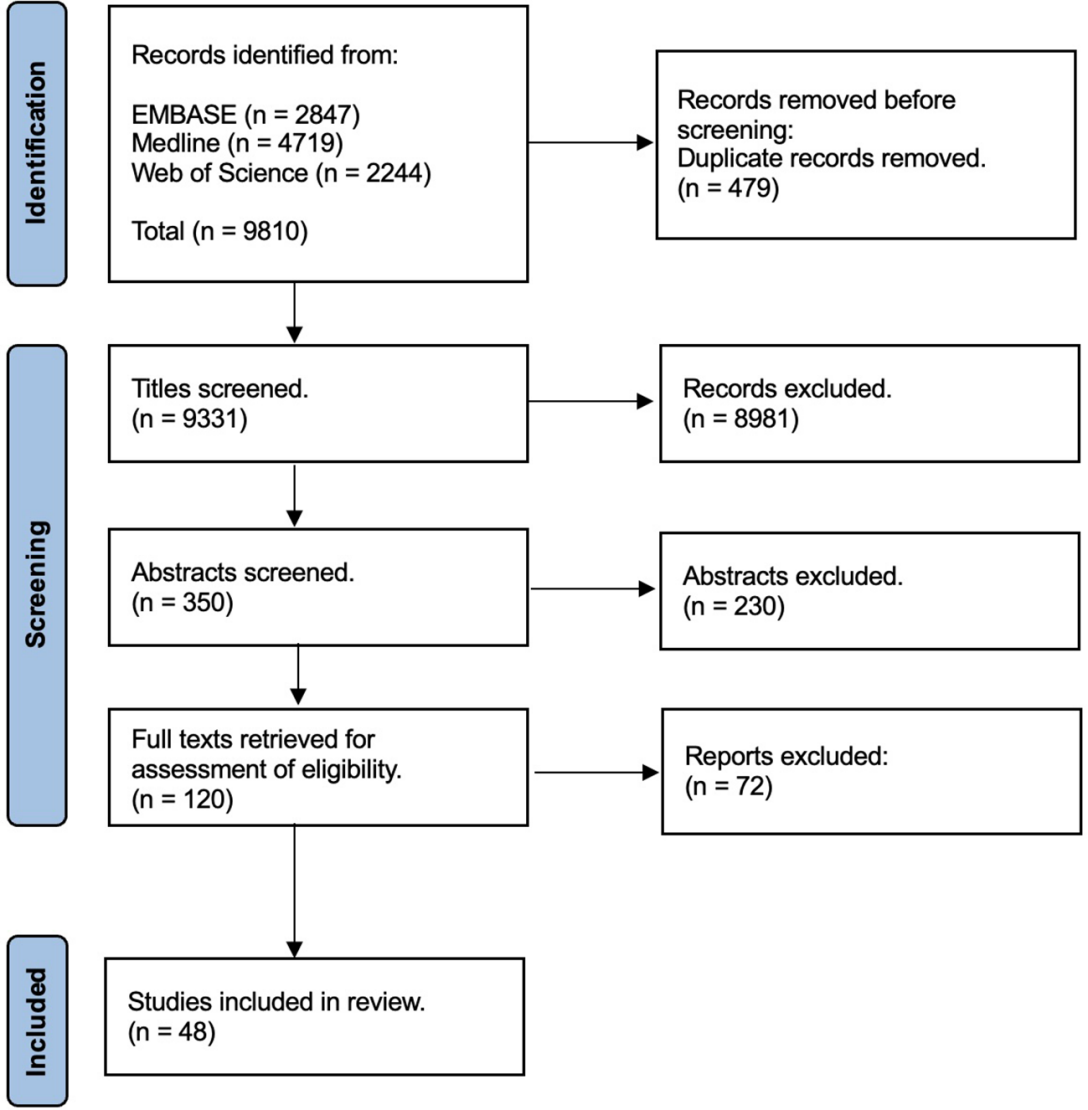
PRISMA flow diagram.

The searches yielded a total of 9,810 publications. When duplicates were removed, there were 9,331 titles to screen. Following title screening there were 350 abstracts to review for inclusion in the scoping review (**Figure 1**).

Following abstract screening there were 120 full papers considered for eligibility for the review. Forty-eight publications were considered to meet the eligibility criteria and therefore included in the final review.

## Summarising the results

Forty-eight publications were selected to inform the review (5, 11–13, 18–61) which included data from nearly 11,000 patients with a diagnosis of cancer (number of HNC cases included in the reviewed publications = 10,808) (**Table 1**).

**Table 1:** Summary of included publications.

Publication	Aims	Location	Dates	Type of publication	Cancer type	Patient numbers (Cancer)	Signs, symptoms & clinical findings related to cancer or the referral criteria for suspected cancer	Conclusions/Limitations/Notable findings
Adham, M.;Kurniawan, A. N.;Muhtadi, A. I.;Roezin, A.;Hermani, B., et al. (18)	Presentation of clinical and epidemiological observations of Indonesian nasopharyngeal cancer patients	Indonesia	1995 to 2005	Retrospective	Nasopharyngeal cancer	1121	Nasal congestion, blood secretion, diplopia, tinnitus, ear problem, cephalgia (head, face, neck pain), unilateral lymph node enlargement, bilateral lymph node enlargement, bilateral lymph node enlargement	Unilateral ear problem, persistent nasal congestion, and nasal blood secretion. A high incidence of nasopharyngeal cancer in Malaysian population
Allgar, V. L.;Oliver, S. E.;Chen, H.;Oviasu, O.;Johnson, M. J., et al. (19)	Improve understanding of the intervals from first symptom recognition to head and neck cancer diagnosis and to investigate the intervals by patient reported symptoms and sociodemographic factors	UK (North-East England)	August 2013 to December 2015	Mixed	Hypopharynx, laryngeal, nasopharyngeal, oral, oropharyngeal, unknown primary - mucosal head and neck cancer	80	Mouth swelling, persistent particularly unilateral pain in the throat (>4/25), painful swallowing, hoarseness, sudden difficulty breathing, weakness or drooping of part of the face, pain, discomfort, unusual sensations in ear, unexplained tooth mobility, other (not specified), difficulty swallowing, neck lump, red/white patch in mouth	33% isolated first symptom, synchronous symptoms common (17% 2 symptoms, 16% 3, 23% 4 or more, 11% no symptoms)
Azhar, N.;Doss, J. G (20).	Explore reasons for advanced stage oral cancer patients delayed health-seeking behaviour and to offer suggestions to improve early cancer diagnosis in a developing country	Malaysia Multisite	3/12 period (no dates)	Qualitative interviews	Oral Cancer	35	Uncomfortable, growth increasing in size, painful, ulcer something whiteish in my mouth, toothache, pain on my gum, gums used to bleed, tooth got loose, discomfort, burning sensation, high fever, ulcer that did not heal and grew quite fast	Translated from Malay - need to create and improve oral cancer awareness amongst Malay population and equip general practitioners with necessary skills to recognise and detect early oral cancer lesions
Aziz, A.;Ramli, R. R.;Mohamad, I.;Bhavaraju, V. M. K (21).	Describe the 8 years’ experience with young nasopharyngeal cancer in a tertiary centre in Malaysia	Malaysia	January 2003 to December 2010	Retrospective	Nasopharyngeal carcinoma	24	Epistaxis, unilateral nasal obstruction, headache, diplopia, unilateral facial paraesthesia, unilateral hearing impairment, tinnitus, otalgia, bilateral nasal obstruction, dysphagia, trismus, unilateral blindness and ptosis, unilateral neck swelling, bilateral neck swelling, ptosis	Most common presenting symptom - neck swelling (45.8% unilateral, 16.7% bilateral - on presentation all had on clinical examination only 70.9% complained of this. 16.6% nasal symptoms, cranial nerve involvement late signs (headache, diplopia, facial paraesthesia)
Bannister, M.;Ah-See, K. W (22).	Contrast features of tonsilitis with oropharyngeal cancer presentation	Scotland	October 2008 to August 2013	Retrospective	Oropharyngeal Carcinoma	18	Sore throat (duration <2 >3 weeks, no fever, non-tender unilateral lymphadenopathy, persistent dysphagia, persistent odynophagia, unilateral otalgia, no symptom improvement with antibiotics, non-tender unilateral lymphadenopathy	Despite smaller number of referrals similar cancer diagnoses made
Bannister, M.;Vallamkondu, V.;Ah-See, K. W (23).	Assess regional incidence, patient profile, tumour site and stage of emergency cancer presentations	Scotland	January 2010 to December 2014	Retrospective	Emergency presentation (thyroid, metastatic to HN from other primary and haematological manifesting in head and neck - excluded)	30	Dysphagia, sore throat, odynophagia, stridor, neck mass, neck mass, stridor	Increasing trend (2.2% proportionately in 2010 to 6.2% in 2014) Increase in oropharyngeal cancers - less dramatic and insidious symptoms compared to laryngeal and oral cavity
Bhamra, N.;Gorman, B.;Arnold, W.;Rajah, A.;Jolly, K., et al. (24)	Investigate impact of coronavirus disease on referral rate of patients to the suspected head and neck two week wait clinic and subsequent management	West Midlands UK (2 centres)	February to April 2019 and February to April 2020	Retrospective	Primary care referrals during Covid – Ear nose and throat and Maxillofacial	509	Unexplained hoarseness >3/52, unexplained persistent sore throat >3/52, pain in head and neck, dysphagia, unexplained nasal obstruction and malodour, mucosal patch with associated pain, swelling, bleeding, unexplained ulceration oral cavity, dental assessment of lump on lip, or oral cavity consistent with oral cancer, red or white patch in oral cavity consistent with erythroplakia or erythroplakia, persistent unexplained neck lump >3/52, unexplained thyroid lump, pain in head/ear with normal otoscopy >4/52, unilateral tonsil swelling, salivary gland swelling	Persistent hoarseness most common referral symptom, highest rate of cancer diagnosis seen in those patients with persistent neck lump
Carpen, T.;Sjoblom, A.;Lundberg, M.;Haglund, C.;Markkola, A., et al. (25)	Characterise the presenting symptoms and clinical findings in patients with HPV positive and negative OPSCC tumours	Finland	February 2012 to 2014	Retrospective	Oropharyngeal carcinoma	118	Neck mass, pain in head and neck region (non-specific- sore throat, odynophagia, ear pain, jaw pain), dysphagia/globus, other - bleeding, weight loss, asymptomatic, neck mass	Neck mass most common presenting symptom, pain more associated with HPV negative OPC reflecting different pathogenesis
Douglas, C. M.;Carswell, V.;Montgomery, J (26).	Determine the outcome of urgent suspicion of cancer referrals for head and neck cancer	Glasgow and Clyde Scotland	June 2015 and May 2016	Retrospective	Primary care referrals include those diagnosed with cancers and non -cancer outcome	235	Hoarseness > 3/52, Cough > 3/52, Neck Lump, odynophagia >3/52, dysphagia > 3/52, Sore throat >3/52, Unilateral sore throat, haemoptysis, globus pharyngeus (not part of referral criteria so specialist determined), oral mucosa or tongue swelling or ulceration, oral mucosa or tongue white/red plaques	Urgent suspicious of cancer pathway 42% patients with head and neck cancer came through this pathway, globus pharyngeus common referral reason despite not being a red flag-frequently referred as dysphagia, referrals cannot be downgraded/rejected
Douglas, C. M.;Ingarfield, K.;McMahon, A. D.;Savage, S. A.;Conway, D. I., et al. (27)	Assess how type and number of symptoms are related to survival in patients with head and neck cancer	Scotland	1 September 1999 to 31 August 2001	Prospective	larynx, oropharynx, oral cavity, and hypopharynx	1589	Hoarseness, pain/discomfort, lump in neck, dysphagia, Ulceration, Swelling, Weight loss, Other unknown	Demonstrates importance of number and type of symptoms in terms of median survival - dysphagia and weight loss - where poor prognostic symptoms at presentation Recommend more rigorous approach to symptom recording using agreed symptom group, reflecting prognosis could be implemented and recorded and the relationship with outcome noted. Is the correct group of symptoms being used in referral guidance and of these symptoms are they sufficiently refined
Douglas, C. M.;Middleton, C.;Sim, P.;Wight, M.;Young, D., et al. (28)	Delineate patterns of hoarseness presentation with a view to assisting referral pathways and whereby reassurance could be provided	Glasgow & Clyde Scotland	April 2015 to April 2016	Retrospective	Persistent hoarseness presentations	61	Persistent hoarseness, intermittent hoarseness (4 cancers), cough, neck lump, odynophagia, Intermittent dysphagia, Persistent dysphagia, Intermittent sore throat, Persistent sore throat, unilateral sore throat, haemoptysis, sensation of a lump in the throat (2 cancers), weight loss, reflux, neck lump, oral mucosa of tongue swelling, oral mucosa red/white patch	Supports an age cut off for referral, inclusion of risk factors like cannabis use (earlier presentation). More questions about hoarseness and its persistence along with demographic data
Esmaelbeigi, F.;Hadji, M.;Harirchi, I.;Omranipour, R.;vand Rajabpour, M., et al. (29)	Study of the situation of professional delays and stage on diagnosis of oral cancer	Iran	2009 to 2010	Questionnaire	Oral cancer	206	Weight loss, otalgia, referred pain, headache, dysphagia, loose teeth, neck lump	Analysis of delay in presentation rather than symptoms patient and physician factors
Fles, R.;Bos, Acrk;Supriyati;Rachmawati, D.;Waliyanti, E., et al. (30)	Explore health behaviours of patients diagnosed with nasopharyngeal cancer and possible causes of patient delay	Indonesia	March 2014 to June 2014	Qualitative	Nasopharyngeal cancer	12	Ringing in one of my ears, headache, runny nose, nose bleeds, double vision, symptoms of the common flu, pain, mass in neck, pain in the bones, dizziness, bronchitis, blood in my sputum	Lack of public knowledge misinterpretation of symptoms, cost a barrier, culture
Ganesan, S.;Sivagnanganesan, S.;Thulasingam, M.;Karunanithi, G.;R, K., et al. (31)	Determine the primary secondary and total diagnostic delay of patients diagnosed with head and neck cancer and to explore the reasons for the delay from the patient perspective	India	2016 to 2017	Qualitative	Oral Cavity, Oropharyngeal, laryngeal	16	Painless swelling, swelling, increasing size of swelling, not able to eat or swallow, pain in the throat, ulcer, burning sensation.	Delays because of symptom appraisal, healthcare costs, poor social support, self-remedy, and self-medication
Gilde, J.;Song, B.;Masroor, F.;Darbinian, J. A.;Ritterman Weintraub, M. L., et al. (32)	Characterise the diagnostic pathway of oropharyngeal carcinoma to identify opportunities for improvement	Northern California USA	1 January 2013 to 31 December 2013	Retrospective	Oropharyngeal Carcinoma	152	Throat pain, bleeding, ear pain, asymptomatic, neck mass, facial/dental/jaw pain, tongue, and salivary gland problems, tonsilitis, neck lump, enlarged tonsils	Oropharyngeal squamous cell carcinoma HPV + tends to present from age 40 and in advanced stages usually clinically silent at primary site and manifests once metastatic to neck (this significantly associated with HPV positivity. Metastatic cervical lymph nodes frequently mistaken as infectious (26% initially prescribed antibiotics)
Hasnaoui, M.;Lahmar, R.;Ben Mabrouk, A.;Masmoudi, M.;Mighri, K., et al. (33)	Compare the clinical presentation and cancer staging between paediatric and adult nasopharyngeal cancer to highlight the main characteristics of the disease within the two subgroups	Tunisia	January 2000 to December 2018	Retrospective	Nasopharyngeal cancer	80	Nasal obstruction, hearing problem, nosebleed, headache, cranial nerve palsies, neck mass, neck mass	Different features between paediatric and adult patients. Neck mass in children. Mostly diagnosed late.
Hu, Z. Z.;Wang, Y (34).	Study the early clinical features of nasal NKTL amongst patients with prominent ocular symptoms and compare them with patients with general nasal NKTL to understand the characteristics of nasal NKTL in central and western China	Zhenghou, China	January 2011 to December 2017	Retrospective	NK/T cell lymphoma - not strictly head and neck cancer but symptoms of presentation may be relevant to head and neck cancer presentation	278	Nasal congestion, purulent/bloody nasal mucus, nose/facial swelling, fever, headache, anosmia, eyelid swelling, eye pain, ocular exophthalmos, epiphora, visual decline/visual field defect.	Early eye symptoms and predominant symptoms, late-stage presentation
Jehannin-Ligier, K.;Dejardin, O.;Lapotre-Ledoux, B.;Bara, S.;Coureau, G., et al. (35)	Determine the characteristics of the patients and their tumours at the time of diagnosis	France	2010	Retrospective	Oral and Oropharyngeal carcinoma	1089	Pain, dyspnoea, dysphonia, oral bleeding, impaired general status, impaired clinical status, neck lump	Presented late stage when symptomatic (severe in 1/3) or visually evident, in half patients’ pain was major symptom.
Joshi, P.;Nair, S.;Chaturvedi, P.;Nair, D.;Agarwal, J. P., et al. (36)	Understand underlying reasons for delay in seeking specialist care by patients with oral cancers	India	2011 to 2012	Questionnaire	Oral Cancer	201	Ulcer, mass, pain, skin involvement	Public and General Practice awareness causing delay
Karatayli-Ozgursoy, S.;Bishop, J. A.;Hillel, A. T.;Akst, L. M.;Best, S. R (37).	Presentation of decade of experience to define clinical presentations and outcomes	John Hopkins, USA	January 2004 to December 2013	Retrospective	Salivary gland Cancers of larynx	6	Dysphagia, dysphonia/hoarseness, haemoptysis, cough	Rare cancer
Karatayli-Ozgursoy, S.;Bishop, J. A.;Hillel, A. T.;Akst, L. M.;Best, S. R (38).	Present experience of a decade with these rare tumours to provide insight into the multimorbidity treatment typically required for long term oncologic success	John Hopkins, USA	January 2004 to December 2013	Retrospective	Non epithelial cancers of larynx	11	Dysphonia, globus, dyspnoea	Rare cancer
Karp, E. E.;Yin, L. X.;Moore, E. J.;Elias, A. J.;O’Byrne, T. J., et al. (39)	Explore both patient and provider factors that increase time from symptom onset to diagnosis in HPV (+) oropharyngeal squamous cell carcinoma	Mayo USA	1 January 2006 to 31 December 2016	Retrospective	Oropharyngeal Carcinoma HPV +	703	Sore throat, dysphagia, otalgia, weight loss, fever, haemoptysis, trismus, neck mass, tonsil mass, base of tongue mass, neck mass, intraoral ulcer, tonsil mass, base of tongue mass	Patient and physician education imperative to decrease delay
Kassirian, S.;Dzioba, A.;Hamel, S.;Patel, K.;Sahovaler, A., et al. (40)	Examine the length and cause of delays in head and neck cancer patient presentation	London Ontario, Canada	September 2017 to September 2018	Mixed	Pan head and neck cancer	102	Mouth sore, change in voice, new skin growth, pain at primary site, bleeding, loose teeth, dentures no longer fitting, trouble opening mouth, trouble chewing, trouble swallowing, pain with swallowing, trouble breathing, headaches, runny nose, stuffy nose, decreased sense of smell, trouble hearing, draining ear, double vision, watery eyes, numbness or tingling, neck mass, white patch, red patch	Does not publish the results of the survey
Khalid, M. B.;Ting, P.;Pai, A.;Russo, J. L.;Bakst, R., et al. (41)	Comprehensively analyse the presenting signs and symptoms of a larger sample of oropharyngeal squamous cell carcinoma to lower the threshold of suspicion for patients who would benefit from a timely referral for HPV + oropharyngeal squamous cell carcinoma	NYC, USA	April 2007 to November 2015	Retrospective	Oropharyngeal squamous cell carcinoma HPV (- & +)	207	Hoarseness, voice changes, hot potato voice, sore throat, dysphagia, otalgia, globus, Other (facial swelling, neck pain, non-specific bleeding, jaw pain, snoring, upper respiratory infection symptoms, mucous production & nasal congestion, weight loss, voice changes, neck mass, oral mass, odynophagia, neck mass, oral mass	Patients may attribute initial symptoms to another more benign cause and presented after a neck mass was present subsequently reporting this as the primary complaint.
Kiessling, S. Y.;Soyka, M. B.;Huber, G. F.;Holzmann, D.;Laske, R. D (42).	Analyse presenting symptoms, the time to correct diagnosis and outcome of a cohort of patients with sino-nasal lymphoma	Zurich, Switzerland	2002 to 2015	Retrospective	Sino-nasal lymphoma (not strictly head and neck cancer but nasal symptoms)	11	Rhinorrhoea, nasal obstruction, epistaxis, touch bleeding, mucopus, weight loss, night sweats, fever, mass in the nose on examination	Bilateral symptoms caused a delay in diagnosis (nasal polyposis) but worse disease specific survival. B symptoms more frequent in those with bilateral disease. Duration to diagnosis influenced by various factors. Rare cancer
Lau, K.;Wilkinson, J.;Moorthy, R (43).	Develop a scoring system that determines the risk of head and neck cancer in a patient to aid GP referrals	Two centres in England (Birmingham and Slough)	1 July 2009 to 1 July 2010 (Birmingham) 1 April 2013–31 August 2013 (Slough)	Statistical modelling	Pan head and neck cancer	73	Unilateral hearing loss, weight loss, dysphagia, odynophagia, neck lump, ear lesion, facial lesion, tongue/oral ulcer, tongue/buccal/oral lesion, thyroid swelling, cheek swelling, unilateral otalgia, parotid gland/submandibular gland mass, dysphonia/hoarse voice, sore throat,	NICE guidelines failed to improve volumes referred or impact on early detection. Need something more sensitive.
Lee, J. J.;Dhepnorrarat, C.;Nyhof-Young, J.;Witterick, I (44).	Evaluation of factors associated with length of delays in diagnosis of head and neck cancer	Toronto, Ontario, Canada	January to July 2013	Qualitative	Pan head and neck cancer (oral cavity, sino-nasal, laryngeal, pharyngeal)	28	Lump or swelling, pain, hoarseness, numbness, sore throat, sores of tongue, difficulty moving jaw, earache, pain, burning, soreness, ulcer, bleeding, red patch, white patch, weight loss, fatigue, night sweats, loosening of teeth.	One surgeons case load - more complex, challenging for dentist and general practice, need for national guidelines, improve training, lack of patient awareness of head and neck cancer
Lim, A. E.;Douglas, C. M.;Montgomery, J (45).	Look at the differences in the length of time to presentation to a specialist clinic and the reasons for delayed presentation to a specialist clinic and the reasons for delayed presentation between head and neck cancer patients and their non-cancer diagnosis counterparts	Glasgow, Scotland	3 month period	Questionnaire	Pan head and neck cancer	23	Throat pain, hoarseness, cough, feeling in throat/foreign body sensation in throat/globus, difficulty swallowing, ear symptoms, lump or ulcer in the mouth, Other (non-red flag symptoms). Neck lump	50% did not fulfil Scottish referral guideline criteria for suspected head and neck cancer, 1/3 delayed presentation because they (patient) were not concerned about symptoms (health literacy), deprivation, alcohol, psychological factors, negative cancer beliefs
Mahalingappa, Y. B.;Khalil, H. S (46).	Audit to compare the presentation, diagnostic work up and treatment outcomes of sino-nasal carcinoma with European position paper on endoscopic management of tumours of nose, paranasal sinuses, and skull base	Plymouth, England	2007 to 2012	Retrospective	Sino-nasal malignancy (squamous cell carcinoma, malignant melanoma, adenocarcinoma, chordoma, neuroblastoma, sarcomatoid carcinoma, Haemangiopericytoma	30	Unilateral nasal blockage, nasal mass, epistaxis, septal ulcer, hard palate lesion, facial numbness, facial pain, swollen eye	Only 23% were two week wait referrals, most presented at late-stage disease, delay in presentation could be attributed to non-specific nature of sino-nasal malignancy symptoms at early stage and to the hidden nature of the mucosal covering of the nose and sinuses making direct visualisation by primary care physicians difficult.
Martin S, Clark SE, Gerrand C, Gilchrist K, Lawal M, Maio L, Martins A, Storey L, Taylor RM, Wells M, Whelan JS, Windsor R, Woodford J, Vindrola-Padros C, Fern LA (47).	Diagnostic pathways for sarcoma and the patient experience	12 hospitals Scotland and England	2017 (Interviews) December to 2021 June 2022 (Secondary analysis)	Mixed retrospective	Sarcoma	9	Pain, lump/swelling, eyes, mouth, jaw	Of sarcomas head and neck ones had the shortest diagnostic interval - rare difficult diagnosis
Martinez-Rodriguez, N.;Dorado, C. B.;Brinkmann, J. C. B.;Ares, M. M.;Alonso, J. S., et al. (48)	Register the most frequently occurring clinical manifestations of maxillary sinus carcinoma in the oral cavity to evaluate clinical staging at the moment of diagnosis and to evaluate treatment efficacy and patient survival rates	Madrid, Spain	2010 to 2016	Retrospective	Maxillary sinus carcinoma	24	Dental mobility, dental displacements, oro-sinus fistula, dental loss, pain, swelling.	Pain or swelling of unknown origin, unexplained widening of periodontal ligament space or dental mobility should be considered warning signs to the dentist
Metcalfe, C.;Dailey, Y.;Lowe, D.;Rogers, S. N (49).	Looks at the effect of the two week wait referrals to one unit following the introduction of the guide and regional educational interventions	Cheshire and Merseyside Local Dental Network, England	3 July to 29 September 2017 1 February to 30 April 2018	Retrospective	Pan head and neck cancer	83	Ulcer, lesion, mass/swelling/lump, white patch	Higher number of referrals from general practitioner versus general dental practitioner, patient education is key aspect in the early diagnosis of mouth cancer in terms of encouraging early presentation to primary care.
Mettias, B.;Charlton, A.;Ashokkumar, S (50).	Exploration of effect of revision of NICE referral guidelines in 2015 on cancer detection rates and compliance with referral guidelines	Leicester, England	January to June 2018	Retrospective	Pan head and neck cancer	66	Persistent hoarseness, sore throat, persistent unilateral sore throat, globus, dysphagia, voice changes, oral swelling, bleeding, earache, neck pain, neck lump, asymmetrical tonsil	2015 would benefit from unilateral sore throat and unilateral otalgia (removed in 2015 revision) and oral bleeding (not included)
Montgomery, J.;Douglas, C. M.;Begbie, F.;Hitchings, A.;MacKenzie, K (51).	Gain knowledge about which symptoms patients have concerns about, and what patients’ expectations of attending an “urgent suspicion of cancer” referral appointment are	Glasgow, Scotland	1 October 2017 to 31 December 2017	Questionnaire	Pan head and neck cancer	17	Neck pain, Cough, ear symptoms, short of breath, non-head and neck symptoms, mucous, nasal symptoms, eye symptoms, blood in saliva, reflux, itch, Globus, throat pain, dysphagia, hoarse, lump/ulcer, other symptoms	Globus is misunderstood, 1/3 patients were not concerned about their symptoms, of cancers diagnosed 1/3 of these patients reported they were not worried about their symptoms - are general practitioners telling them its suspected cancer referral. Lack of performance of suspected head and neck cancer clinic.
Nieminen, M.;Aro, K.;Makitie, A.;Harlin, V.;Kainulainen, S., et al. (52)	Hypothesised that due to the rarity of head and neck cancer their diagnostics in primary health care is difficult. Aimed to provide a rough estimate for the likelihood that a primary health care patient of a certain age with a specific symptom has head and neck cancer	Helsinki, Finland	2016	Retrospective	Pan head and neck cancer	40	Lump in the head and neck site, non-healing ulcers, red or white patches in the mouth, painful swallowing, pain in the throat, difficulty swallowing, blocked nose, bloody discharge from nose, difficulties breathing, haemoptysis, hoarseness.	For every head and neck cancer encountered in primary care a general practitioner will meet 6000 patients 100 of whom will have a sign or symptoms potentially caused by head and neck cancer
Queenan, J. A.;Gottlieb, B. H.;Feldman-Stewart, D.;Hall, S. F.;Irish, J., et al. (53)	Investigates patients’ symptom appraisal, help seeking and lay consultancy up to the time they first went to see a health care professional	Kingston & Toronto, Ontario, Canada	September 2010 to December 2011 (Kingston) November 2010 to December 2011 (Toronto)	Qualitative	Oral cavity, oropharynx, larynx	83	Mouth lesion, pain, neck mass, voice/speech problem, problems swallowing, bleeding, loose dentures/tooth, sinus problems, facial swelling, taste changes, plugged ear, other (not specified)	About symptom appraisal suggests need deeper understanding of how or if the symptom appraisal and help seeking processes affect the time from first symptom recognition to contacting a health care practitioner.
Raman, A.;Sen, N.;Ritz, E.;Fidler, M. J.;Revenaugh, P., et al. (54)	1) Further characterise the presentation and diagnosis of HPV-associated OPSCC, and 2) Determine if the variability in presentation and diagnosis of oropharyngeal squamous cell carcinoma within an HPV-positive patient population impacts times to presentation, diagnosis, and treatment initiation	Chicago, USA	2008 to 2018	Retrospective	Oropharyngeal carcinoma HPV +	84	Primary site symptoms only, neck mass plus primary site symptoms, no symptoms, sore throat, dysphagia, odynophagia, ear pain bleeding, asymptomatic neck mass	Heterogenous presentation, asymptomatic neck mass may delay but equally symptomatic without a neck mass might delay imaging
Roennegaard, A. B.;Rosenberg, T.;Bjorndal, K.;Sorensen, J. A.;Johansen, J., et al. (5)	1) Present set up of the Head and neck cancer fast track programme 2) Present patient characteristics, diagnostic outcome, cancer detection rate and duration of the fast-track patient courses	Odense University Hospital, Denmark	1 July 2012 to 1st September 2015	Prospective	Pan head and neck cancer	1285	Bloody nasal secretion, recurrent nasal haemorrhage, nasal wounds, tumour in nasal cavity, Unilateral secretory otitis media, affection of cranial nerves, wounds in oral cavity or oropharynx, tumour in oral cavity or oropharynx, pain radiation to ear, enlarged submandibular lymph node, hoarseness, difficulty swallowing/globulus, tumour in salivary gland, growth of known salivary gland tumour, tumour in salivary gland with simultaneous affection of the facial nerve, tumour in the thyroid gland with simultaneous hoarseness, rapid growth of known thyroid gland, hard immobile tumour in the thyroid gland, enlarged lymph nodes with no infectious or benign cause, a lateral neck cyst in patients more than 40 years of age, Unilateral nasal stenosis	Head and neck cancer detection 29.2%
Rovira, A.;Brar, S.;Munroe-Gray, T.;Ofo, E.;Rodriguez, C., et al. (55)	Analyse the outcomes of telephone consultation including patient satisfaction for two week wait HNC referrals	London, England	23 March 2020 to 19 June 2020	Prospective	Pan head and neck cancer	6	Mouth ulcer, tongue ulcer, palate lump, sore or painful throat, neck mass, hoarseness, tinnitus, thyroid, facial pain, nasal mass, nasal discharge, tonsil mass, tonsil asymmetry, hearing loss	Hoarseness and presence of neck lump (thyroid lymph node salivary gland) more difficult to assess than sore throat remotely
Shephard, E. A.;Parkinson, M. A.;Hamilton, W. T (56).	Identify and quantify the laryngeal cancer risk for individual and combined clinical features (symptoms, physical signs, and abnormal investigations) of primary care patients	England	2000 to 2009	Statistical modelling	laryngeal cancer	806	Hoarseness, sore throat, chest infection (a composite), dysphagia, otalgia, dyspnoea, insomnia, mouth symptoms, raised inflammatory markers.	Importance of combinations of symptoms and second presentation with same symptoms
Storck, K.;Brandstetter, M.;Keller, U.;Knopf, A (57).	Analysis of characteristics of histologically defined head and neck lymphoma to raise awareness of ENT specialists to the leading symptoms	Munich, Germany	January 2003 to December 2011	Retrospective	Lymphoma in the head and neck region	221	Cervical neck mass, odynophagia, dysphagia, globus pharyngeus, dysphonia, dyspnoea	Only 13% of those diagnosed with lymphoma presented with constitutional or specific B symptoms (weight loss, fever, night sweats), can be a challenge
Talwar, C.;McClune, A.;Kelly, D.;Lowe, D.;Rogers, S. N (58).	Reports on urgent two week wait referrals from primary care to highlight the difficulties with the two week wait referral route	Liverpool, England	January 2017 to January 2019	Retrospective	Pan head and neck cancer - patients referred under TWW	6	Neck lump, oral lesion, dysphagia, throat pain, neck pain, mass on imaging, facial swelling, chest infection, ear pain, asymmetrical tonsils, chronic cough, sore tongue, dysphagia, weight loss, change in voice, white patch on throat, oral thrush, parotid swelling, hoarseness.	Suggest that combination of symptoms is a stronger indication of malignancy, as a single symptom however persistent of unexplained hoarseness warrants concern - better proforma would help secondary care triage
Tikka, T.;Pracy, P.;Paleri, V (11).	Identify and refine the set of referral criteria that will provide optimal diagnostic efficacy using data obtained from a large cohort of patients who were referred using the previous iteration of fast track criteria with a suspected head and neck cancer by applying well defined statistical techniques	Birmingham & Newcastle Upon Tyne, England	January 2007 to December 2010 (Newcastle Upon Tyne) July 2009 to July 2010 (Birmingham)	Statistical modelling	Pan head and neck cancer	230	Unexplained neck mass >3 weeks, hoarseness >3 weeks, sensation of a lump in throat, intermittent hoarseness, orbital mass, blood in mouth, otalgia, oral ulceration >3weeks, odynophagia >3weeks, cranial neuropathies, oral lip mass >3 weeks, intermittent dysphagia, lip lesion, persistent sore throat/painful throat, red and white patches or oral mucosa, unexplained tooth mobility not associated with periodontal disease >3 weeks, unexplained otalgia with normal otoscopy, unexplained persistent ear lesion, unexplained sore nose, unilateral nasal obstruction particularly with associated with purulent discharge, unilateral unexplained pain in the head and neck area >4 weeks	Persistence and laterality important in sore throat, and relevant to dysphagia, neck lump, hoarseness
Tikka, T.;Paleri, V.;MacKenzie, K (12).	To validate the head and neck cancer risk calculator using a cohort of patients urgently referred with suspected head and neck cancer	Glasgow, Scotland	June 2015 to May 2016	Statistical modelling	Pan head and neck cancer	232	Unexplained neck mass >3 weeks, hoarseness >3 weeks, sensation of a lump in throat, intermittent hoarseness, orbital mass, blood in mouth, otalgia, oral ulceration >3weeks, odynophagia >3weeks, cranial neuropathies, oral lip mass >3 weeks, intermittent dysphagia, lip lesion, persistent sore throat/painful throat, red and white patches or oral mucosa, unexplained tooth mobility not associated with periodontal disease >3 weeks, unexplained otalgia with normal otoscopy, unexplained persistent ear lesion, unexplained sore nose, unilateral nasal obstruction particularly with associated with purulent discharge, unilateral unexplained pain in the head and neck area >4 weeks, unintentional weight loss	External validation inclusion of social factors including alcohol and smoking status
Tikka, T.;Kavanagh, K.;Lowit, A.;Pan, J. F.;Burns, H., et al. (13)	To further increase the predictive power of the HaNC-RC by assessing the potential for inclusion of other significant symptoms, the refinement of symptoms already in the model and the addition of social history factors	Glasgow, Scotland	January 2017 to December 2018	Statistical modelling	Pan head and neck cancer	397	Unintentional weight loss, hoarseness, sore throat, throat discomfort/irritation, feeling of something in the throat (FOSIT), Dysphagia, Regurgitation, Odynophagia, Neck pain, Neck lump, choking episode/feeling, catarrh/mucus, blocked nose, oral swelling, oral ulcer, haemoptysis, unexplained otalgia with normal otoscopy, face pain/numbness, noisy breathing/stridor, red/white patch in mouth, persistent head dan neck skin lesion	Excluded symptoms which presented in less than 10 patients. Call for refining of referral criteria. To improve diagnostic efficacy. Missing data so smoking history not included
Venchiarutti, R. L.;Pho, L.;Clark, J. R.;Palme, C. E.;Young, J. M (59).	Investigate the patient and carer perceptions of facilitators and barriers to early diagnosis of head and neck cancer	NSW, Australia	April 2019 to May 2020	Qualitative	Pan head and neck cancer	39	Lump, numbness, tingling, change in snoring (noted by partner), sore tooth, solid lump, swollen ear, lump in throat, neck lump.	Complex nature of factors facilitating and impeding early head and neck cancer diagnosis and treatment.
Wang, K. H.;Austin, S. A.;Chen, S. H.;Sonne, D. C.;Gurushanthaiah, D (60).	Diagnostic time intervals, presenting symptoms, diagnostic accuracy of nasal endoscopy, imaging, and diagnosis at first otolaryngologist visit	Kaiser Permanente Northern California	1 January 2007 to 31 December2010	Retrospective	Nasopharyngeal carcinoma	101	Hearing loss, otitis media, headache, cranial neuropathy, diplopia, facial numbness, facial droop, tongue numbness, nasal obstruction, epistaxis, neck mass, sinusitis type symptoms	Often multiple symptoms, can remain clinically silent for a long period of time, 1/3 patients had symptoms from multiple categories and 42 different symptom combinations were seen
Williams, C.;Byrne, R.;Holden, D.;Sherman, I.;Srinivasan, V. R (61).	Analyse trends in two week rule referrals for head and neck cancer over 10 years - audit	Wirral, Merseyside, England	1 January 2002 to 31 December 2002 1 January 2012 to 30 June 2012	Retrospective	Pan head and neck cancer	31	Hoarseness, neck lump, unexplained persistent sore throat, dysphagia, persistent swelling salivary gland, unilateral nasal obstruction, oral swelling, ulceration of oral mucosa, unhealing ulcer or skin lesion, orbital mass, red or white patches, ulcerated or pigmented skin lesions, unilateral pain in the head and neck area, thyroid.	12% conversion rate, referral rate increasing disproportionately to the cancer diagnoses, modifications should be made to improve the quality of patient care and decrease the pressure of these referrals on Ear nose and throat departments

Most of the publications present European data (5, 11–13, 19, 22–28, 35, 42, 43, 45–48, 50–52, 55–58, 61) of which the majority were from the UK (11–13, 19, 22–24, 26–28, 43, 45–47, 49–51, 55, 56, 58, 61). Eight of the selected papers were from lower or middle income countries (18, 20, 21, 30, 31, 33, 34, 36), seven from the USA (32, 37–39, 41, 54, 60), three from Canada (40, 44, 53) and one from Australia (59).

The majority of the papers selected were retrospective studies using data from patient records. These explored characteristics of a cohort or case series of patients with a particular HNC (for example nasopharyngeal carcinoma (54, 60), HPV positive oropharyngeal carcinoma) or a group of cancers arising in the head and neck [for example a cohort including oral, oropharynx, larynx and hypopharyngeal (27)] (11–13, 18, 21–26, 32–35, 37, 38, 41–43, 46, 48, 50, 52, 54, 56–58, 60, 61).

Seventeen papers (5, 11–13, 24, 26, 28, 43–45, 49–52, 55, 58, 61) included retrospective analysis of patients referred on a suspected HNC referral pathway; most of these were from the UK (England TWW (11, 24, 43, 49, 50, 55, 58, 61) or Scotland USOC route (12, 13, 26, 28, 45, 51). Three were from outside the UK (4, 44, 52). These papers included analysis of the signs, symptoms, and risk factors more common amongst the patients who were diagnosed with HNC.

Of the retrospective cohort series reports most were from the UK. Five studies used data to statistically model the presenting signs and symptoms with which patients are referred to specialist care for suspected cancer, and defined the symptoms associated with a diagnostic outcome of a HNC (11–13, 43, 49, 56). These papers aimed for a clinically applicable risk stratification system which might be used to make decisions on urgency of referrals or to triage referrals. There was only one prospective study from Denmark (5), arising from the HNC fast track referral programme in Odense.

There were six qualitative studies (20, 30, 31, 44, 53, 59), all of which were from outside the UK. These explored the patient and health system factors related to the delays in presentation and diagnosis associated with HNCs (20, 30, 31, 44, 59). One explored patient symptom appraisal (53) and its role in timing of presentation to health care services.

Four selected articles presented the results of patient questionnaires, three explored factors determining delay in presentation and diagnosis (29, 36, 45), and one was concerned with patient expectations of a suspected cancer referral and appointment (51).

Three papers used a non-traditional mixed method approach (19, 40, 47), two using retrospective notes examination with patient questionnaires (19, 40) [one from the UK (19)] and a third included data from patient interviews (47) from a UK sarcoma cohort which included 9 patients diagnosed with a head and neck sarcoma. All aimed to explore the reasons behind delay in diagnosis and presentation.

Some papers referred specifically to a type of HNC, often rare like sino-nasal (46, 48) and nasopharyngeal carcinoma (18, 21, 30, 33, 60); a number of studies concentrated on HPV positive and negative oropharyngeal cancer (22, 25, 32, 39, 41, 54) and oral cancers (20, 29, 36), with one including both (35). Studies on multiple HNC subtypes based on suspected cancer referrals often had a small minority with diagnosed cancer (5, 11–13, 24, 26, 28, 43–45, 50–52, 55, 58, 61). Three studies were based on laryngeal cancers (37, 38, 56), two of which were rare types of laryngeal cancers [non-epithelial (37) and salivary gland cancers of the larynx (38)]. The selected data included papers on some cancers which are not regarded as HNCs in the traditional sense but which frequently present with important symptoms related to the function and anatomy of the head and neck e.g. lymphoma (34, 42, 57) and sarcoma (47). These patients are likely to present/be referred to ENT for clinical assessment in the first instance.

The aims of the studies can be categorised into the following: explorations for the reasons for delay in diagnosis or presentation (19, 20, 22, 23, 29–32, 36, 39, 40, 44, 45, 47, 59, 60), the relationship between presenting symptoms and outcomes (18, 21, 25–28, 37, 38, 42, 46, 48, 49, 51–54, 56, 57), the predictive factors (including symptoms and risk factors) for an eventual diagnosis of a HNC (33–35, 41), development of statistical models to assist referrals for suspected cancer (11–13, 43). A small number of the published papers present an assessment of the fast track referral systems for suspected cancer (5, 24, 50, 55, 58, 61) (only one from outside the UK (5)).

When it comes to the symptoms of HNC in these papers, it is important to consider the perspective and the language. There are five papers where the data is based on primary care coding or referral category (5, 22, 52, 56, 58); in others, the presenting symptoms and referral criteria from primary care have been used to explore the strength of the relationship between various signs and symptoms and a cancer outcome.

When the selected studies discussed symptoms, these tended to be in terms of medical language (5, 11–13, 18, 21–24, 28, 29, 32–35, 38–40, 42–44, 48, 53, 54, 56, 58, 61) with some including clinical signs or findings and other using diagnostic terminology but referring to it in the same category as a symptom (25–27, 36, 37, 41, 45, 46, 49, 50, 52, 55, 57, 60).

The patient perspective and language is presented in just seven papers (20, 30, 31, 47, 51, 53, 59). Five papers focus on the language used by patients to describe their head and neck symptoms (20, 30, 31, 47, 59). One paper used medical terminology to discuss patient level of concern (51) the other grouped patient symptoms into medicalised groups (for example voice/speech problem and pain or bleeding (53).

In those papers which present a retrospective assessment of the clinical notes the authors fail to clarify whether data included objective examination findings noted by the specialist and diagnoses which a primary care clinician had not made or included as part of their referral (globus as opposed to dysphagia and the physical presence or absence of a neck lump for example) (11–13, 24).

Because most the publications included in this scoping review were internal departmental retrospective case note reviews there is no external funding reported. Where there is funding declared it is all from academic non-commercial research grants (20, 29–31, 53, 56).

## The symptoms of head and neck cancer

The extracted data related to signs, symptoms and referral criteria were categorised in terms of the type of language: patient or lay terms for symptoms, clinical terms for symptoms and the language of clinical examination findings, signs, and diagnostic terms. The results are displayed in **Table 2** and are divided into the anatomical areas of the head and neck which the symptoms affect along with some of the systemic symptoms associated with cancers in general derived from the literature. **Table 2** includes columns which contains the site to which symptoms are related the physical signs which patients may complain of and the medical language of both signs and symptoms.

**Table 2:** Summary of symptoms, presenting complaints, clinical findings and medical language of head and neck cancer.

Site (Often significant because of symptoms’ persistence and unilaterality)	Patient Symptom (may complain of physical signs with symptoms related to a physical change)	Physical signs on examination and/or the location of the symptoms	Medical Language (symptoms)
**Mouth**	Sore tongue Tongue numbness Tongue swelling Mouth swelling Mouth sore Burning Blood in saliva Blood in mouth Discomfort Toothache Gum pain Tingling Taste changes	Dentures not fitting Loose tooth Associated neck or oral cavity/mouth/lip/palate/gum/mucosa/tongue/periodontal, lump, mass, enlargement, swelling, lymph node, tumour lesion, ulcer, white patch, red patch, plaque, abnormality, fast growing, non-healing Oral thrush	Paraesthesia
**Throat**	Choking episode/feeling Difficulty swallowing Feeling in the throat Feeling of something in the throat Pain with swallowing Painful swallowing Sensation of lump in the throat Sore throat Painful throat Throat discomfort/irritation Trouble swallowing Snoring Change in voice Regurgitation	Associated throat/tonsil/pharynx/base of tongue or neck, lump, mass, enlargement, lymph node, swelling, ulcer, patch, lesion, abnormality, tumour Asymmetry/Asymmetrical Unilateral Bilateral	Foreign body sensation in throat Regurgitation Dysphagia Odynophagia Reflux Globus sensation Hot potato voice
**Nose**	Blocked nose Catarrh/mucus Decreased sense of smell Malodour Mucus production Nasal congestion Nasal discharge Nasal obstruction Nose bleed Stuffy nose Bloody discharge from the nose Sinusitis Runny nose	Nasal wounds Septal ulcer Sinus fistula Nasal stenosis Associated neck lump, mass, enlargement, lesion, swelling, lymph node, ulcer, patch, lesion, abnormality, tumour Purulent Skin lesion	Epistaxis Anosmia Rhinorrhoea Nasal Haemorrhage Paraesthesia Mucopus Sinusitis symptoms
**Ear**	Draining ear Ear pain Earache Hearing loss Hearing impairment Trouble hearing Unusual sensations in ear Tinnitus Ringing of one of my ears Dizziness Plugged ear	Normal otoscopy Otitis Media Secretory otitis media Associated neck or, lump, mass, enlargement, swelling, lymph node, ulcer, patch, lesion, abnormality	Tinnitus Hearing impairment Otalgia Paraesthesia Radiation to ear
**Neck**	Pain	Associated neck or primary site lump, mass, enlargement, swelling, lymph node, ulcer, patch, lesion, abnormality Thyroid gland, salivary gland, submandibular, parotid, lateral neck cyst Mass Enlargement Unilateral Lump – a physical sign may be asymptomatic (no symptoms associated with the lump)/non tender	Asymptomatic
**Head\Face**	Face pain Numbness Headache Weakness of part of the face Tingling	Facial droop Drooping of part of the face Associated neck or primary site, lump, mass, enlargement, swelling, lymph node, lesion, patch, ulcer, abnormality Cranial nerve palsies Cranial neuropathies Ulcerated or pigmented skin lesions	Cephalgia
**Eyes**	Blindness Double vision Eye pain Visual decline Watery eyes	Swollen lid Swollen eye Associated neck or primary site, lump, mass, enlargement, swelling, lymph node, lesion, ulcer, patch, abnormality Eyelid swelling Orbital mass Ptosis Exophthalmos	Diplopia Epiphora
**Trachea/Larynx**	Cough Difficulties breathing Noisy breathing Short of breath Sudden difficulty breathing Trouble breathing Blood in my sputum	Associated neck or primary site, lump, mass, enlargement, swelling, lymph node, lesion, ulcer, patch, abnormality	Dyspnoea Stridor Haemoptysis
**Larynx specific to voice**	Change in voice Hoarseness Voice changes Voice/speech problem	Associated neck or primary site, lump, mass, enlargement, swelling, lymph node, lesion, ulcer, patch, abnormality	Dysphonia
**Jaw**	Difficulty moving jaw Jaw pain Dental pain Loosening of teeth Mobile tooth Trouble chewing Trouble opening mouth	Associated neck or primary site, lump, mass, enlargement, swelling, lymph node, lesion, ulcer, patch, abnormality Dental mobility Dental displacement Dental loss	Trismus
**General** **(local or distant to primary cancer/constitutional)**	Discomfort Soreness Painful Tingling Numbness Weakness Fatigue Fever Itch Night sweats Weight loss	Skin growth Associated neck or primary site, lump, mass, enlargement, swelling, lesion, ulcer, patch, abnormality	Unintentional weight loss Impaired general status Insomnia

In the literature, signs are sometimes conflated with symptoms such as a mouth ulcer or a neck lump, these are objective clinical findings. A patient may notice an asymptomatic neck or salivary gland lump and this might be the reason they present to primary care or a specialist (where there is no gatekeeper system), however, the assumption is that if a patient is referred from primary care there is, on clinical examination, a palpable neck or salivary gland lump. The term symptom is sometimes used in the reviewed literature when the language refers to a clinically determined diagnosis such as globus pharyngeus.

Despite some of the symptoms being rarely associated with an eventual diagnosis of cancer (fewer than those with a benign cause), some patients will experience and describe these symptoms in the journey to their cancer diagnosis and so these are included in **Table 2**.

## Discussion

This scoping review aimed to produce a comprehensive collection of terminology in the literature related to the symptoms associated with HNC patient presentations. Forty-eight publications were identified, and the data extracted has been presented, organised according to anatomical and functional sites of the head and neck, in terms of patient and clinical language of the symptoms and those which are considered a presenting complaint which is subject to an objective clinical examination. This summary will be used and expanded upon in the EVEREST-HN project with the aim of harnessing the patient narrative in estimating risk of undiagnosed HNC.

The language pertaining to the symptoms of HNC is important because successful diagnosis depends on effective communication regarding signs and symptoms between patients and clinicians and between different health care professionals: unfortunately, what a term like odynophagia means to a specialist is different to that which a GP might understand (62). It is also true that how a patient communicates symptoms can be re/mis-interpreted by a GP leading to over or under emphasising the clinical significance (63). UK ENT surgeons complain that some primary care clinicians misinterpret a neck lump as a lump in the throat when a patient has no palpable neck lump (64). An ulcer is a physical sign found on examination and can be associated with symptoms like bleeding or pain but findings in the mouth might be interpreted differently by a GDP compared to a GP or a nurse practitioner with no dental experience. Fear of missing a cancer may drive referrals along with lack of relevant clinical experience, pressures related to capacity to book follow up appointments, continuity of care and difficulty accessing alternatives to the urgent cancer pathway (such as dental care and routine hospital appointments).

There are important issues drawn out of this literature review in terms of what is considered a sign and what is a symptom. When patients present to primary care they do not say “I have been experiencing hoarseness, haemoptysis, dyspnoea, odynophagia, unilateral sore throat with ipsilateral otalgia, with normal otoscopy and level IV cervical lymphadenopathy”; instead, they might say “my throat has been sore for weeks and my ear hurts on the same side, I can’t swallow my food properly so am having soups, my voice is croaky and my breathing is noisy”. They might say “there’s a painful white patch on the roof of my mouth which has been bleeding” as opposed to “leukoplakia of the hard palate” and a primary care clinician may not have the confidence to describe it as such either.

In the UK, odynophagia, dysphagia, and hoarseness are the commonest “symptoms” for which patients are referred to ENT from GPs and yet very few of these patients will be diagnosed with a cancer (conversion from referral to cancer diagnosis of less than 3% (7). The fact that GPs are referring patients with these symptoms because they suspect a HNC implies that they do not have the confidence to clinically reassure themselves or patients that an underlying cancer does not exist. For some GPs, excluding a cancer may necessitate a referral much to the chagrin of head and neck surgeons (both ENT and OMFS) many of the swallowing issues (referred as dysphagia and odynophagia) are given a benign diagnosis of globus pharyngeus it is also notable that these symptoms no longer appear on the NICE referral criteria for suspected HNC (are part of upper gastrointestinal pathway) yet some English Cancer Alliances have retained these symptoms on their local referral proformas. A recent review of the diagnosis and investigation of globus by the joint European and American Society of Neurogastroenterology and Motility describe it as a “clinical challenge” diagnosed “when the clinical history is supportive and after alternate medical conditions have been excluded using prudent and specific investigation” (65) such expertise and appropriate investigations are not routinely accessible, without significant delay, to primary care in the UK.

Triage of suspicious oral lesions seen by primary care (GPs), for which NICE recommend a general dental opinion (increasingly difficult to access in the NHS) prior to a TWW referral from GPs, might be facilitated using teledentistry alongside clinical history and physical examination performed in the community by both GPs and GDPs (66). Teledentistry could improve communication between primary care and secondary care and provide a timely expert opinion about the route of referral and or appropriate management in the community, this is particularly pertinent given the current crisis in dental care provision in the UK (8). It could certainly be an adjunct to the patient reported symptom questions proposed by the EVEREST-HN programme for lesions visible in the mouth and oropharynx.

The HaNC-RC work and retrospective studies into HNC patients highlights that there are combinations of risk factors, symptoms and signs which make a cancer more likely to be causing symptoms. The sources exploring the links between signs and symptoms and an outcome of a diagnosis of HNC are mostly from secondary care and predominantly from UK sources (two authors dominate these sources Tikka and Douglas). It appears an almost uniquely UK preoccupation with the sensitivity of the referrals and compliance with the clinical criteria pertaining to these referrals. The reasons for this are justified by the poor yield of cancer from this referral route (7) and demands on secondary care services to meet the NHS Faster Diagnosis Framework Pathway Standard (67). Publications from other countries are more concerned with reasons for late presentation, delayed diagnosis, and patient symptom appraisal. When it comes to HNC, the suspected cancer referral routes in both England and Scotland are a source of frustration to secondary care as its existence does not appear to be affecting the yield or the treatment outcomes of HNCs.

Remote triage using HaNC-RC was not a perfect tool even in the hands of senior specialists (68) and when used by GPs increased rather than decreased the volumes referred (69). EVEREST-HN aims to further explore these relationships and potentially integrate digital images and voice recordings to develop a system which stratifies patients in a way that benefits the NHS, the patients and cancer outcomes better than the current system.

## Limitations

Because of resources and time constraints it was not possible to complete grey searches or to gather international clinical guidelines and HNC referral criteria. Other sources such as patient facing cancer websites and patient forums would be another source of valuable data in terms of the lay perspective and language around the symptoms of HNC.

This review has not subdivided the nomenclature of the symptoms and signs of HNC into head and neck site specific areas but rather into function and anatomical areas. There is undoubtedly cross over between symptoms which occur in several subsites where cancers of the head and neck originate which will sometimes depend on the stage of the cancer at which the patient presents because it may invade local tissues affecting function, EVEREST-HN aspires to expose some of these relationships with the aim to utilise this information for future triaging of referrals.

The review includes symptoms of rare HNCs and the more common ones including oral, oropharyngeal, and laryngeal cancers. EVEREST-HN will gather data on the rare and the common symptoms and identify ways in which these can be stratified into high and low risk using the patient reported symptoms so that the vast majority can be investigated or assessed quickly by the most appropriate clinician. EVEREST-HN will capture data to use in the future to target questions about symptoms, signs, and risk factors to triage more effectively those most likely to have cancer to appropriate services with the aim to improve the patient journey (for the vast majority without a cancer and those with an underlying cancer) and best optimise the finite NHS resources.

## Conclusions

Symptoms of HNC are common presenting complaints in primary care, interpreting these along with clinical history, examination and risk factors will inform a clinician’s decision to refer as suspected cancer. The TWW suspected cancer referral criteria have evolved over the years as specialists consider a different way of triaging the volume of referrals that is needed to assess the clinical risk of an undiagnosed HNC over and above the referral based on primary care assessment. EVEREST-HN aims to achieve this using the patient history of their symptoms. This review has highlighted issues in terms of what is considered a symptom, a presenting complaint and a clinical finding or sign. Difficulties remain in terms of interpretation, of the language of signs and symptoms and the physical findings, by both patients as well as generalists. The way patients report their own symptoms will be crucial along in combination with other modalities like digital photography, voice recordings and the potential of artificial intelligence, to develop a more a more sensitive triage system.

## Data availability statement

The raw data supporting the conclusions of this article will be made available by the authors, without undue reservation.

## Author contributions

PB: Conceptualization, Data curation, Formal analysis, Investigation, Methodology, Project administration, Writing – original draft, Writing – review & editing. YL: Data curation, Writing – review & editing. AA: Writing – review & editing. JH: Writing – review & editing. IK: Writing – review & editing. CO: Writing – review & editing. RR: Writing – review & editing. NR: Writing – review & editing. TT: Writing – review & editing. JP: Writing – review & editing. VP: Writing – review & editing

## EMBASE

1. “head and neck neoplasms”/or “squamous cell carcinoma of head and neck”/or mouth neoplasms/or gingival neoplasms/or lip neoplasms/or palatal neoplasms/or salivary gland neoplasms/or parotid neoplasms/or sublingual gland neoplasms/or submandibular gland neoplasms/or tongue neoplasms/or otorhinolaryngologic neoplasms/or ear neoplasms/or laryngeal neoplasms/or nose neoplasms/or paranasal sinus neoplasms/or pharyngeal neoplasms/or hypopharyngeal neoplasms/or nasopharyngeal neoplasms/or nasopharyngeal carcinoma/or oropharyngeal neoplasms/or tonsillar neoplasms/or thyroid neoplasms/or thyroid cancer, papillary/.
2. “predictive value of tests”/or roc curve/.
3. diagnosis/or clinical decision-making/or delayed diagnosis/or “early detection of cancer”/.
4. “Referral and Consultation”/mt, td [Methods, Trends].
5. clinical audit/or dental audit/or medical audit/or practice guidelines as topic/.
6. Symptom Assessment/sn [Statistics & Numerical Data].
7. 2 or 3 or 4 or 5 or 6.
8. 1 and 7.
9. limit 8 to yr=“2012 -Current”.

## MEDLINE

1. (Head and Neck Neoplasms).mp. [mp=title, book title, abstract, original title, name of substance word, subject heading word, floating sub-heading word, keyword heading word, organism supplementary concept word, protocol supplementary concept word, rare disease supplementary concept word, unique identifier, synonyms].
2. Otorhinolaryngologic Neoplasms.mp. or Otorhinolaryngologic Neoplasms/.
3. exp Mouth Neoplasms/di [Diagnosis].
4. Thyroid Neoplasms/or Thyroid Carcinoma, Anaplastic/or Carcinoma/.
5. Pharyngeal Neoplasms.mp. or Pharyngeal Neoplasms/.
6. Laryngeal Neoplasms.mp. or Laryngeal Neoplasms/.
7. Tongue Neoplasms.mp. or Tongue Neoplasms/.
8. Salivary Gland Neoplasms.mp. or Salivary Gland Neoplasms/.
9. Oropharyngeal Neoplasms.mp. or Oropharyngeal Neoplasms/.
10. (Squamous Cell Carcinoma of Head and Neck).mp. [mp=title, book title, abstract, original title, name of substance word, subject heading word, floating sub-heading word, keyword heading word, organism supplementary concept word, protocol supplementary concept word, rare disease supplementary concept word, unique identifier, synonyms].
11. Paranasal Sinus Neoplasms.mp. or Paranasal Sinus Neoplasms/.
12. Maxillary Sinus Neoplasms.mp. or Maxillary Sinus Neoplasms/.
13. Nose Neoplasms.mp. or Nose Neoplasms/.
14. 1 or 2 or 3 or 4 or 5 or 6 or 7 or 8 or 9 or 10 or 11 or 12 or 13.
15. oropharyngeal cancer.mp. [mp=title, book title, abstract, original title, name of substance word, subject heading word, floating sub-heading word, keyword heading word, organism supplementary concept word, protocol supplementary concept word, rare disease supplementary concept word, unique identifier, synonyms].
16. (head and neck cancer).mp. [mp=title, book title, abstract, original title, name of substance word, subject heading word, floating sub-heading word, keyword heading word, organism supplementary concept word, protocol supplementary concept word, rare disease supplementary concept word, unique identifier, synonyms].
17. (head and neck cancer).mp. [mp=title, book title, abstract, original title, name of substance word, subject heading word, floating sub-heading word, keyword heading word, organism supplementary concept word, protocol supplementary concept word, rare disease supplementary concept word, unique identifier, synonyms].
18. mouth cancer.mp. [mp=title, book title, abstract, original title, name of substance word, subject heading word, floating sub-heading word, keyword heading word, organism supplementary concept word, protocol supplementary concept word, rare disease supplementary concept word, unique identifier, synonyms].
19. thyroid cancer.mp. [mp=title, book title, abstract, original title, name of substance word, subject heading word, floating sub-heading word, keyword heading word, organism supplementary concept word, protocol supplementary concept word, rare disease supplementary concept word, unique identifier, synonyms].
20. pharyngeal cancer.mp. [mp=title, book title, abstract, original title, name of substance word, subject heading word, floating sub-heading word, keyword heading word, organism supplementary concept word, protocol supplementary concept word, rare disease supplementary concept word, unique identifier, synonyms].
21. pharynx cancer.mp. [mp=title, book title, abstract, original title, name of substance word, subject heading word, floating sub-heading word, keyword heading word, organism supplementary concept word, protocol supplementary concept word, rare disease supplementary concept word, unique identifier, synonyms].
22. tongue cancer.mp. [mp=title, book title, abstract, original title, name of substance word, subject heading word, floating sub-heading word, keyword heading word, organism supplementary concept word, protocol supplementary concept word, rare disease supplementary concept word, unique identifier, synonyms].
23. salivary gland cancer.mp. [mp=title, book title, abstract, original title, name of substance word, subject heading word, floating sub-heading word, keyword heading word, organism supplementary concept word, protocol supplementary concept word, rare disease supplementary concept word, unique identifier, synonyms].
24. parotid cancer.mp. [mp=title, book title, abstract, original title, name of substance word, subject heading word, floating sub-heading word, keyword heading word, organism supplementary concept word, protocol supplementary concept word, rare disease supplementary concept word, unique identifier, synonyms].
25. submandibular cancer.mp. [mp=title, book title, abstract, original title, name of substance word, subject heading word, floating sub-heading word, keyword heading word, organism supplementary concept word, protocol supplementary concept word, rare disease supplementary concept word, unique identifier, synonyms].
26. (Squamous Cell Carcinoma head and neck).mp. [mp=title, book title, abstract, original title, name of substance word, subject heading word, floating sub-heading word, keyword heading word, organism supplementary concept word, protocol supplementary concept word, rare disease supplementary concept word, unique identifier, synonyms].
27. oropharyngeal cancer.mp. [mp=title, book title, abstract, original title, name of substance word, subject heading word, floating sub-heading word, keyword heading word, organism supplementary concept word, protocol supplementary concept word, rare disease supplementary concept word, unique identifier, synonyms].
28. sinus cancer.mp. [mp=title, book title, abstract, original title, name of substance word, subject heading word, floating sub-heading word, keyword heading word, organism supplementary concept word, protocol supplementary concept word, rare disease supplementary concept word, unique identifier, synonyms].
29. paranasal sinus cancer.mp. [mp=title, book title, abstract, original title, name of substance word, subject heading word, floating sub-heading word, keyword heading word, organism supplementary concept word, protocol supplementary concept word, rare disease supplementary concept word, unique identifier, synonyms].
30. maxillary sinus cancer.mp. [mp=title, book title, abstract, original title, name of substance word, subject heading word, floating sub-heading word, keyword heading word, organism supplementary concept word, protocol supplementary concept word, rare disease supplementary concept word, unique identifier, synonyms].
31. nose cancer.mp. [mp=title, book title, abstract, original title, name of substance word, subject heading word, floating sub-heading word, keyword heading word, organism supplementary concept word, protocol supplementary concept word, rare disease supplementary concept word, unique identifier, synonyms].
32. 15 or 16 or 17 or 18 or 19 or 20 or 21 or 22 or 23 or 24 or 25 or 26 or 27 or 28 or 29 or 30 or 31.
33. 14 or 32.
34. (Signs and Symptoms).mp. [mp=title, book title, abstract, original title, name of substance word, subject heading word, floating sub-heading word, keyword heading word, organism supplementary concept word, protocol supplementary concept word, rare disease supplementary concept word, unique identifier, synonyms].
35. Predictive Value of Tests.mp. or “Predictive Value of Tests”/.
36. Diagnosis/or Diagnosis.mp.
37. Early Diagnosis.mp. or Early Diagnosis/.
38. Physical Examination.mp. or Physical Examination/.
39. Diagnosis, Oral.mp. or Diagnosis, Oral/.
40. (Referral and Consultation).mp. [mp=title, book title, abstract, original title, name of substance word, subject heading word, floating sub-heading word, keyword heading word, organism supplementary concept word, protocol supplementary concept word, rare disease supplementary concept word, unique identifier, synonyms].
41. 34 or 35 or 36 or 37 or 38 or 39 or 40.
42. 33 and 41.
43. Quality Improvement/or Medical Audit/or Clinical Audit Clinical Audit Clinical Audit.mp.
44. guideline.mp. or Guideline/.
45. 43 or 44.
46. 42 and 45.
47. Logistic Models.mp. or Logistic Models/.
48. 33 and 47.
49. 46 or 48.

## Web of Science

1. TS=(prediction) 1,545,739.
2. TS=(signs symptoms) 94,033.
3. TS=(early diganosis) 2.
4. #1 OR #2 OR #3 1,638,130.
5. head and neck cancer (Topic) 79,998.
6. #4 AND #5 1,922.
